# Association of Psychiatric Disorders With Incidence of SARS-CoV-2 Breakthrough Infection Among Vaccinated Adults

**DOI:** 10.1001/jamanetworkopen.2022.7287

**Published:** 2022-04-14

**Authors:** Kristen Nishimi, Thomas C. Neylan, Daniel Bertenthal, Karen H. Seal, Aoife O’Donovan

**Affiliations:** 1 Mental Health Service, San Francisco Veterans Affairs Health Care System, San Francisco, California; 2 Department of Psychiatry and Weill Institute for Neurosciences, University of California San Francisco, San Francisco; 3 Integrative Health Service, San Francisco Veterans Affairs Health Care System, San Francisco, California; 4 Department of Medicine, University of California, San Francisco

## Abstract

**Importance:**

Psychiatric disorders may be associated with an increased risk for SARS-CoV-2 breakthrough infection after vaccination, but no studies have tested this hypothesis.

**Objective:**

To evaluate whether past diagnoses of psychiatric disorders are associated with an increased incidence of SARS-CoV-2 breakthrough infection among fully vaccinated individuals.

**Design, Setting, and Participants:**

This retrospective cohort study included data from the administrative and electronic health records of US Department of Veterans Affairs (VA) patients from February 20, 2020, to November 16, 2021. Participants included 263 697 patients who accessed VA health care during the study period, had at least 1 SARS-CoV-2 test recorded in the electronic health record, had no record of SARS-CoV-2 infection prior to vaccination, and had completed a full SARS-CoV-2 vaccination regimen 14 days or more prior.

**Exposures:**

Psychiatric disorder diagnoses in the past 5 years, including depressive, posttraumatic stress, anxiety, adjustment, alcohol use, substance use, bipolar, psychotic, attention-deficit/hyperactivity, dissociative, and eating disorders.

**Main Outcomes and Measures:**

SARS-CoV-2 breakthrough infections, defined as positive SARS-CoV-2 tests, among fully vaccinated individuals.

**Results:**

Of 263 697 fully vaccinated VA patients (239 539 men [90.8%]; mean [SD] age, 66.2 [13.8] years), 135 481 (51.4%) had at least 1 psychiatric disorder diagnosis, and 39 109 (14.8%) developed a breakthrough infection. A diagnosis of any psychiatric disorder was associated with increased incidence of breakthrough infection, both in models adjusted for potential confounders (adjusted relative risk [aRR], 1.07; 95% CI, 1.05-1.09) and additionally adjusted for medical comorbidities and smoking (aRR, 1.03; 95% CI, 1.01-1.05). Most specific psychiatric disorder diagnoses were associated with an increased incidence of breakthrough infection, with the highest relative risk observed for adjustment disorder (aRR, 1.13; 95% CI, 1.10-1.16) and substance use disorders (aRR, 1.16; 95% CI, 1.12-1.21) in fully adjusted models. Stratifying the sample at 65 years of age revealed that associations between psychiatric diagnoses and incident breakthrough infection were present in both age groups but were stronger and robust to adjustment for medical comorbidities and smoking among older patients.

**Conclusions and Relevance:**

This cohort study suggests that psychiatric disorder diagnoses were associated with an increased incidence of SARS-CoV-2 breakthrough infection among VA patients, with the strongest associations observed for older individuals. Individuals with psychiatric disorders may be at heightened risk for contracting COVID-19 even after vaccination, suggesting the need for targeted prevention efforts.

## Key Points

**Question:**

Are psychiatric disorders associated with an increased risk for SARS-CoV-2 breakthrough infection after vaccination?

**Findings:**

In this cohort study of 263 697 fully vaccinated US Department of Veterans Affairs patients, psychiatric disorder diagnoses were associated with increased incidence of SARS-CoV-2 breakthrough infection after vaccination.

**Meaning:**

This study suggests that targeted strategies for preventing SARS-CoV-2 breakthrough infections should be considered for individuals with psychiatric disorders.

## Teaser

This cohort study uses data from US Department of Veteran Affairs patients to evaluate whether past diagnoses of psychiatric disorders are associated with an increased incidence of SARS-CoV-2 breakthrough infection among fully vaccinated individuals.

## Introduction

Early efforts to disseminate vaccines against COVID-19 were moderately successful in the US, with 68.8% of individuals aged 12 years or older in the general population being fully vaccinated as of November 2021 and an estimated 63.8% of US Department of Veterans Affairs (VA) patients fully vaccinated as of October 2021.^1,2^ However, given waning immunity, incomplete immunization coverage, and variants that exhibit resistance to vaccine-induced neutralizing antibodies, SARS-CoV-2 breakthrough infections are relatively common and play an important role in prolonging the pandemic.^1,2^ Prior to the widespread availability of vaccinations, individuals with psychiatric disorders were at heightened risk for contracting COVID-19 and for experiencing severe COVID-19 sequelae, including hospitalization and death.^3,4,5,6^ Thus, there is a need to identify whether psychiatric disorders increase the risk for SARS-CoV-2 breakthrough infections after vaccination so that targeted preventive interventions (eg, booster shots and public health campaigns) can be used in this population if warranted.

Several factors may be associated with the increased risk for SARS-CoV-2 breakthrough infection among individuals with psychiatric disorders. First, studies have demonstrated impaired immune function and poor response to vaccines among individuals with psychiatric disorders,^7,8,9,10^ raising the possibility of reduced immunity after vaccination against SARS-CoV-2 in this group.^2,11,12^ Second, emerging data indicate that individuals with psychiatric disorders may engage in more risky behaviors for contracting SARS-CoV-2,^13^ which in turn may play a key role in determining risk for COVID-19, even after vaccination.^14,15^ Third, medical conditions, such as cardiovascular disease, diabetes, and chronic obstructive pulmonary disease, and deleterious behaviors, such as smoking, are more common among individuals with psychiatric disorders^16,17,18^ and have been identified as risk factors for SARS-CoV-2 breakthrough infection among VA patients.^11,12^ Despite these lines of evidence, only 1 study reported that individuals with substance use disorders were at increased risk for SARS-CoV-2 breakthrough infection,^19^ and we lack information on the risk for contracting breakthrough infection among those with other psychiatric disorders.

In the present study, we examined associations between psychiatric disorders and the incidence of SARS-CoV-2 breakthrough among fully vaccinated VA patients. Our central hypothesis was that psychiatric disorders would be associated with an increased incidence of SARS-CoV-2 breakthrough infection. Because older individuals are vulnerable to COVID-19, in general,^20^ and show the greatest losses in immunity against SARS-CoV-2,^21^ we performed secondary analyses that stratified the cohort at 65 years of age to evaluate whether associations between psychiatric disorders and breakthrough infections differed by age group. Prior studies among VA patients have examined waning vaccine effectiveness over time^2^ and identified younger age, White vs Black race, and Hispanic or Latinx ethnicity as risk factors for SARS-CoV-2 breakthrough infection,^11,12^ although, to our knowledge, no studies have examined associations of psychiatric disorders with incident SARS-CoV-2 breakthrough infection among VA patients.

## Methods

### Study Design and Participants

This retrospective cohort study included 263 697 individuals who sought VA health care nationwide between February 20, 2020, and November 16, 2021, had at least 1 positive or negative SARS-CoV-2 test recorded in VA clinical notes, and were fully vaccinated against SARS-CoV-2 after December 1, 2020. To derive the analytic sample, we identified 1 629 439 VA patients who accessed VA health care, had a SARS-CoV-2 test, and had no record of a SARS-CoV-2 infection prior to their vaccination (because a prior SARS-CoV-2 infection among vaccinated individuals lowers the risk for a breakthrough infection).^22^ From these patients, we excluded 49 052 patients who had not used VA health care in the 12 months before their SARS-CoV-2 test; 1 313 794 pateints who did not have a SARS-CoV-2 vaccination recorded in VA records or were not fully vaccinated before November 16, 2021; 525 patients who had improbably early vaccine dates (before December 2020), indicating errors in their records; 65 patients who received a SARS-CoV-2 vaccine type other than BNT162b2, mRNA-1273, or JNJ-78436735; 200 patients who had hospital admissions more than 5 days before their recorded SARS-CoV-2 test; and 2106 patients who were missing data on body mass index, a key covariate. Full vaccination was defined as occurring at least 14 days after 2 doses of messenger RNA vaccine (ie, Pfizer-BioNTech or Moderna) or 1 dose of Johnson & Johnson–Janssen vaccine, consistent with the Centers for Disease Control and Prevention definition.^14^ Data came from the VA Corporate Data Warehouse, a regional database of VA patient data and electronic health records, and the VA COVID-19 Shared Data Resource, a database of all patients with a SARS-CoV-2 test recorded in VA clinical notes. Our study was reported in accordance with the Strengthening the Reporting of Observational Studies in Epidemiology (STROBE) guideline.^23^ This study was approved by the Committee on Human Research, University of California, San Francisco, and the San Francisco VA Health Care System Human Research Protection Program, and a waiver of informed consent was approved for analysis of records data.

### Measures

Psychiatric disorders included diagnoses of depressive disorder, posttraumatic stress disorder, anxiety disorders, adjustment disorder, alcohol use disorder, substance use disorders, bipolar disorders, psychotic disorder, attention-deficit/hyperactivity disorder, dissociative disorders, and eating disorder, identified with *International Classification of Diseases, Ninth Revision, Clinical Modification* (*ICD-9-CM*) or *International Statistical Classification of Diseases and Related Health Problems, Tenth Revision, Clinical Modification* (*ICD-10-CM*) codes from inpatient or outpatient clinical data in the past 5 years (eTable 1 in the Supplement). Breakthrough infections were defined as a positive SARS-CoV-2 test recorded in VA clinical notes (among individuals ≥14 days after their final SARS-CoV-2 vaccine dose). Covariates included age, sex (male or female), race (Black or African American, White, other [American Indian or Alaska Native, Asian, and Native Hawaiian or Other Pacific Islander], or unknown race), ethnicity (Hispanic or Latinx, not Hispanic or Latinx, or unknown ethnicity), medical conditions (clinical diagnoses of any of the following in the past 2 years: diabetes, cardiovascular disease including hypertension, obstructive sleep apnea, cancer, chronic obstructive pulmonary disease, chronic kidney disease, liver disease, and HIV), obesity (defined as body mass index ≥35 [calculated as weight in kilograms divided by height in meters squared]), and smoking status (current or former smoker or never smoker), all derived from administrative data or *ICD-9-CM* or *ICD-10-CM* codes in the electronic health record. Race and ethnicity information was derived from self-reports by patients to VA staff.^24^

### Statistical Analysis

Generalized linear models with Poisson distribution and log link for relative risks (RRs)^25^ with robust error variance estimated associations between diagnoses of psychiatric disorders and incidences of breakthrough infection, including an offset parameter to account for participants’ time at risk. Model 1 adjusted for potential confounders, including sociodemographic factors (ie, age, sex, and race and ethnicity), vaccine type, and time since vaccination (including an interaction term for vaccine type by time since vaccination to account for differential waning effectiveness).^21^ Model 2 additionally adjusted for medical conditions, obesity, and smoking because these concurrent health-related factors may represent confounders or possible mediators of the association between psychiatric disorders and incident SARS-CoV-2 breakthrough infection. Models were performed first for any psychiatric disorders vs none and then for each specific individual disorder vs none in secondary analyses. We defined *any psychiatric disorder diagnosis* using all the included psychiatric disorders, but we conducted specific disorder models only for disorders with a prevalence in the full sample of 2.5% or more; therefore, individual models were not assessed for attention-deficit/hyperactivity disorder (1.6% [4262]), dissociative disorders (0.3% [760]), or eating disorders (0.3% [698]).

In additional secondary analyses, we reran all models in samples stratified at 65 years of age. As a sensitivity analysis, we performed the primary models with a more conservative definition of breakthrough infections—positive SARS-CoV-2 tests at least 30 days after receipt of full vaccination regimen—to limit misclassification of breakthroughs due to infection acquired before vaccination.^14^ In another sensitivity analysis, we performed the primary models by excluding 6256 patients who received a booster vaccination (ie, >6 months after final Moderna dose, >5 months after final Pfizer-BioNTech dose, or >60 days after Johnson & Johnson–Janssen dose) to evaluate whether booster vaccines were associated with the findings. Data were prepared with SAS, version 9.4 (SAS Institute Inc) and analyzed with Stata, version 15.1 (StataCorp LLC). All *P* values were from 2-sided tests and results were deemed statistically significant at *P* < .05.

## Results

Of 263 697 fully vaccinated VA patients who met inclusion criteria (mean [SD] age, 66.2 [13.8] years; 239 539 men [90.8%]; 54 168 Black or African American patients [20.5%], 21 770 Hispanic or Latinx patients [8.3%], 184 901 White patients [70.1%], and 24 628 patients of other or unknown race [9.3%]), 135 481 (51.4%) had at least 1 psychiatric disorder diagnosis in the past 5 years (Table 1). Breakthrough infection occurred in 14.8% of the sample (39 109). Relative to patients with no psychiatric disorders, those with any psychiatric disorder had a 7% higher incidence of breakthrough infection (adjusted RR [aRR], 1.07; 95% CI, 1.05-1.09; *P* < .001), adjusted for potential confounders (Table 2). Estimates were attenuated by approximately 3.7% but remained significant when additionally adjusted for medical conditions, obesity, and smoking (aRR, 1.03; 95% CI, 1.01-1.05; *P* < .001).

**Table 1.** Distribution of Covariates Among 263 697 Fully Vaccinated VA Patients

Covariate	VA patients, No. (%)			*P* value
	Full sample (N = 263 697)	No psychiatric disorders (n = 128 216 [48.6%])	Any psychiatric disorder (n = 135 481 [51.4%])	
Age, mean (SD), y	66.2 (13.8)	70.1 (12.3)	62.5 (14.1)	<.001
Sex				
Female	24 158 (9.2)	7002 (5.5)	17 156 (12.7)	<.001
Male	239 539 (90.8)	121 214 (94.5)	118 325 (87.3)	
Race				
Black or African American	54 168 (20.5)	22 940 (17.9)	31 228 (23.0)	<.001
White	184 901 (70.1)	93 403 (72.8)	91 498 (67.5)	
Other or unknown^a^	24 628 (9.3)	11 873 (9.3)	12 755 (9.4)	
Ethnicity				
Not Hispanic or Latinx	229 809 (87.1)	112 864 (88.0)	116 945 (86.3)	<.001
Hispanic or Latinx	21 770 (8.3)	8500 (6.6)	13 270 (9.8)	
Unknown	12 118 (4.6)	6852 (5.3)	5266 (3.9)	
Obesity (BMI ≥35)	48 668 (18.5)	21 476 (16.7)	27 192 (20.1)	<.001
Medical comorbidities				
Diabetes	96 004 (36.4)	49 161 (38.3)	46 843 (34.6)	<.001
Cardiovascular disease	94 247 (35.7)	48 906 (38.1)	45 341 (33.5)	<.001
Obstructive sleep apnea	81 684 (31.0)	31.050 (24.2)	50 634 (37.4)	<.001
Cancer	60 685 (23.0)	32 147 (25.1)	28 538 (21.1)	<.001
COPD	45 819 (17.4)	21 500 (16.8)	24 319 (18.0)	<.001
Chronic kidney disease	38 822 (14.7)	21 132 (16.5)	17 690 (13.1)	<.001
Liver disease	17 463 (6.6)	6970 (5.4)	10 493 (7.7)	<.001
HIV	2294 (0.9)	888 (0.7)	1406 (1.0)	<.001
Smoking				
Current or former smoker	169 143 (64.1)	82 167 (64.1)	86 976 (64.2)	<.001
Never smoker	94 554 (35.9)	46 049 (35.9)	48 505 (35.8)	

Abbreviations: BMI, body mass index (calculated as weight in kilograms divided by height in meters squared); COPD, chronic obstructive pulmonary disease; VA, US Department of Veterans Affairs.

^a^ Other race includes American Indian or Alaska Native, Asian, and Native Hawaiian or Other Pacific Islander.

**Table 2.** Associations Between Psychiatric Disorders and SARS-CoV-2 Breakthrough Infections Among Fully Vaccinated VA Patients, in the Full Sample and Age Stratified

Psychiatric disorder	VA Patients, No. (%)	Model 1^a^		Model 2^b^	
		RR (95% CI)	*P* value	RR (95% CI)	*P* value
Full sample (N=263 697)					
Any psychiatric disorder	135 481 (51.4)	1.07 (1.05-1.09)	<.001	1.03 (1.01-1.05)	<.001
Major depressive disorder	84 588 (32.1)	1.11 (1.09-1.13)	<.001	1.05 (1.03-1.08)	<.001
Posttraumatic stress disorder	61 674 (23.4)	1.08 (1.05-1.10)	<.001	1.03 (1.01-1.06)	.006
Anxiety disorder	53 988 (20.5)	1.13 (1.11-1.16)	<.001	1.08 (1.06-1.11)	<.001
Adjustment disorder	28 352 (10.8)	1.18 (1.15-1.22)	<.001	1.13 (1.10-1.16)	<.001
Alcohol use disorder	22 942 (8.7)	1.06 (1.03-1.10)	<.001	1.05 (1.02-1.09)	.002
Substance use disorder	14 089 (5.3)	1.18 (1.14-1.23)	<.001	1.16 (1.12-1.21)	<.001
Bipolar disorder	9439 (3.6)	1.12 (1.07-1.18)	<.001	1.07 (1.02-1.12)	.004
Psychotic disorder	7326 (2.8)	1.08 (1.02-1.14)	.009	1.05 (0.99-1.11)	.09
Age <65 y (n = 97 972)					
Any psychiatric disorder	64 251 (65.6)	1.03 (1.00-1.07)	.03	1.00 (0.97-1.03)	.82
Major depressive disorder	43 217 (44.1)	1.06 (1.02-1.09)	.001	1.01 (0.98-1.05)	.41
Posttraumatic stress disorder	30 212 (30.8)	1.06 (1.03-1.10)	.001	1.03 (1.00-1.07)	.09
Anxiety disorder	31 023 (31.7)	1.08 (1.04-1.12)	<.001	1.04 (1.00-1.08)	.01
Adjustment disorder	16 544 (16.9)	1.13 (1.09-1.18)	<.001	1.09 (1.05-1.14)	<.001
Alcohol use disorder	13 137 (13.4)	1.05 (1.00-1.10)	.04	1.04 (0.99-1.09)	.11
Substance use disorder	8745 (8.9)	1.12 (1.07-1.18)	<.001	1.11 (1.05-1.17)	<.001
Bipolar disorder	6073 (6.2)	1.05 (0.99-1.12)	.09	1.01 (0.95-1.08)	.68
Psychotic disorder	3553 (3.6)	0.92 (0.85-1.00)	.06	0.90 (0.82-0.97)	.009
Age ≥65 y (n = 165 725)					
Any psychiatric disorder	71 203 (43.0)	1.10 (1.07-1.13)	<.001	1.05 (1.03-1.08)	<.001
Major depressive disorder	41 358 (25.0)	1.15 (1.12-1.19)	<.001	1.08 (1.05-1.11)	<.001
Posttraumatic stress disorder	31 458 (19.0)	1.08 (1.04-1.11)	<.001	1.03 (1.00-1.06)	.09
Anxiety disorder	22 956 (13.9)	1.18 (1.14-1.22)	<.001	1.12 (1.09-1.16)	<.001
Adjustment disorder	11 803 (7.1)	1.21 (1.16-1.27)	<.001	1.14 (1.10-1.19)	<.001
Alcohol use disorder	9804 (5.9)	1.06 (1.00-1.11)	.04	1.05 (1.00-1.11)	.06
Substance use disorder	5344 (3.2)	1.27 (1.20-1.36)	<.001	1.24 (1.16-1.32)	<.001
Bipolar disorder	3365 (2.0)	1.22 (1.13-1.32)	<.001	1.16 (1.07-1.25)	<.001
Psychotic disorder	3773 (2.3)	1.26 (1.17-1.36)	<.001	1.23 (1.15-1.33)	<.001

Abbreviations: RR, relative risk; VA, US Department of Veterans Affairs.

^a^ Model 1: age, age squared, sex, race, ethnicity, vaccine type, time since vaccination, and vaccine type × time since vaccination. Reference group is no psychiatric disorders.

^b^ Model 2: model 1 plus obesity status, diabetes, cardiovascular disease including hypertension, obstructive sleep apnea, chronic obstructive pulmonary disease, cancer, chronic kidney disease, liver disease, HIV, and smoking. Reference group is no psychiatric disorders.

Across specific psychiatric disorders, each was associated with an increased incidence of breakthrough infection (Table 2) among the full sample in models adjusted for confounders. Estimates were attenuated in magnitude but remained significant for most individual disorders when additionally accounting for medical conditions, obesity, and smoking (Figure). Adjustment disorder (aRR, 1.13; 95% CI, 1.10-1.16; *P* < .001) and substance use disorders (aRR, 1.16; 95% CI, 1.12-1.21; *P* < .001) were associated with the highest increase in incident breakthrough infection, and only psychotic disorders were no longer associated with an increased incidence of breakthrough infection after additional adjustment (aRR, 1.05; 95% CI, 0.99-1.11; *P* = .09).

**Figure.**
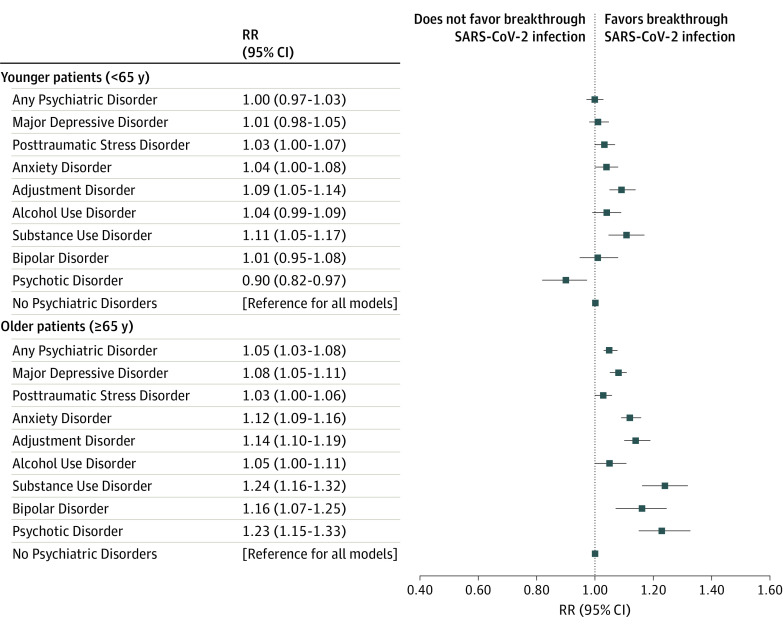
Relative Risks (RRs) of SARS-CoV-2 Breakthrough Infection for Individual Psychiatric Disorders Stratified by Age The reference group for each model is no psychiatric disorders; each individual psychiatric disorder was estimated in a separate model as the primary factor and adjusted for age, age squared, sex, race, ethnicity, vaccine type, time since vaccination, vaccine type × time since vaccination, obesity status, diabetes, cardiovascular disease including hypertension, obstructive sleep apnea, chronic obstructive pulmonary disease, cancer, chronic kidney disease, liver disease, HIV, and smoking.

Younger age was associated with a higher incidence of breakthrough infection (association with 5-year change in age in model 1: aRR, 0.93; 95% CI, 0.92-0.95; *P* < .001) (Table 3), with 15 615 of 97 972 patients (15.9%) younger than 65 years and 23 472 of 165 725 patients (14.2%) 65 years or older experiencing a breakthrough infection. Psychiatric disorders were also more common among younger patients than older patients (any psychiatric disorder: 64 251 of 97 972 [65.6%] vs 71 203 of 165 725 [43.0%]).

**Table 3.** Associations Between Psychiatric Disorders and SARS-CoV-2 Breakthrough Infections Among Fully Vaccinated VA Patients, Including Covariates

Characteristic	Model 1^a^		Model 2^b^	
	RR (95% CI)	*P* value	RR (95% CI)	*P* value
Full sample (N = 263 697)				
Any psychiatric disorder	1.07 (1.05-1.09)	<.001	1.03 (1.01-1.05)	.001
Age (per 5-y change)	0.93 (0.92-0.95)	<.001	0.88 (0.86-0.90)	<.001
Age squared	1.00 (1.00-1.00)	<.001	1.00 (1.00-1.00)	<.001
Male (vs female)	1.05 (1.01-1.08)	.004	1.00 (0.97-1.03)	.90
Black or African American (vs White)	0.81 (0.79-0.83)	<.001	0.81 (0.79-0.83)	<.001
Other or unknown race (vs White)^c^	0.94 (0.91-0.97)	<.001	0.95 (0.92-0.98)	.002
Hispanic or Latinx (vs not Hispanic or Latinx)	0.90 (0.87-0.93)	<.001	0.90 (0.87-0.93)	<.001
Time since vaccination	0.82 (0.81-0.83)	<.001	0.82 (0.81-0.83)	<.001
Moderna vaccine	0.16 (0.15-0.17)	<.001	0.16 (0.14-0.17)	<.001
Pfizer-BioNTech vaccine	0.24 (0.22-0.26)	<.001	0.24 (0.22-0.26)	<.001
Johnson & Johnson–Janssen vaccine	1 [Reference]	NA	1 [Reference]	NA
Moderna vaccine × time since vaccination	1.24 (1.22-1.26)	<.001	1.24 (1.22-1.26)	<.001
Pfizer-BioNTech vaccine × time since vaccination	1.20 (1.18-1.22)	<.001	1.20 (1.18-1.22)	<.001
Johnson & Johnson–Janssen vaccine × time since vaccination	1 [Reference]	NA	1 [Reference]	NA
Diabetes	NA	NA	1.10 (1.08-1.12)	<.001
Cardiovascular disease	NA	NA	1.19 (1.17-1.22)	<.001
Obstructive sleep apnea	NA	NA	1.13 (1.10-1.15)	<.001
Cancer	NA	NA	1.07 (1.04-1.09)	<.001
COPD	NA	NA	1.18 (1.15-1.20)	<.001
Chronic kidney disease	NA	NA	1.23 (1.20-1.26)	<.001
Liver disease	NA	NA	1.11 (1.07-1.14)	<.001
HIV	NA	NA	1.20 (1.10-1.31)	<.001
Obesity	NA	NA	1.10 (1.08-1.13)	<.001
Current or former smoker (vs never smoker)	NA	NA	0.95 (0.93-0.97)	<.001
Age <65 y (n = 82 180)				
Any psychiatric disorder	1.03 (1.00-1.07)	.03	1.00 (0.97-1.03)	.82
Age (per 5-y change)	0.98 (0.97-0.99)	<.001	0.95 (0.94-0.96)	<.001
Male (vs female)	1.04 (1.00-1.08)	.04	1.00 (0.96-1.04)	.92
Black or African American (vs White)	0.86 (0.83-0.89)	<.001	0.84 (0.81-0.87)	<.001
Other or unknown race (vs White)^c^	0.92 (0.88-0.97)	.001	0.93 (0.89-0.97)	.002
Hispanic or Latinx (vs not Hispanic or Latinx)	0.01 (0.96-1.06)	.69	1.00 (0.95-1.04)	.88
Time since vaccination	0.81 (0.79-0.82)	<.001	0.81 (0.79-0.82)	<.001
Moderna vaccine	0.18 (0.16-0.20)	<.001	0.18 (0.16-0.20)	<.001
Pfizer-BioNTech vaccine	0.26 (0.24-0.29)	<.001	0.26 (0.24-0.29)	<.001
Johnson & Johnson–Janssen vaccine	1 [Reference]	NA	1 [Reference]	NA
Moderna vaccine × time since vaccination	1.22 (1.19-1.25)	<.001	1.22 (1.19-1.24)	<.001
Pfizer-BioNTech vaccine × time since vaccination	1.19 (1.16-1.21)	<.001	1.19 (1.16-1.21)	<.001
Johnson & Johnson–Janssen vaccine × time since vaccination	1 [Reference]	NA	1 [Reference]	NA
Diabetes	NA	NA	1.10 (1.06-1.13)	<.001
Cardiovascular disease	NA	NA	1.22 (1.18-1.27)	<.001
Obstructive sleep apnea	NA	NA	1.11 (1.08-1.14)	<.001
Cancer	NA	NA	1.11 (1.07-1.16)	<.001
COPD	NA	NA	1.20 (1.14-1.26)	<.001
Chronic kidney disease	NA	NA	1.23 (1.17-1.30)	<.001
Liver disease	NA	NA	1.13 (1.08-1.19)	<.001
HIV	NA	NA	1.19 (1.06-1.33)	.003
Obesity	NA	NA	1.09 (1.06-1.13)	<.001
Current or former smoker (vs never smoker)	NA	NA	0.94 (0.91-0.97)	<.001
Age ≥65 y (n = 142 208)				
Any psychiatric disorder	1.10 (1.07-1.13)	<.001	1.05 (1.03-1.08)	<.001
Age (per 5-y change)	1.02 (1.01-1.03)	<.001	1.01 (1.00-1.02)	.009
Male (vs female)	1.11 (1.04-1.18)	.003	1.04 (0.98-1.11)	.21
Black or African American (vs White)	0.77 (0.74-0.79)	<.001	0.77 (0.74-0.79)	<.001
Other or unknown race (vs White)^c^	0.95 (0.91-0.99)	.03	0.97 (0.93-1.02)	.23
Hispanic or Latinx (vs not Hispanic or Latinx)	0.78 (0.74-0.82)	<.001	0.80 (0.76-0.84)	<.001
Time since vaccination	0.84 (0.82-0.86)	<.001	0.84 (0.82-0.86)	<.001
Moderna vaccine	0.15 (0.14-0.18)	<.001	0.15 (0.13-0.17)	<.001
Pfizer-BioNTech vaccine	0.23 (0.20-0.26)	<.001	0.23 (0.20-0.26)	<.001
Johnson & Johnson–Janssen vaccine	1 [Reference]	NA	1 [Reference]	NA
Moderna vaccine × time since vaccination	1.23 (1.20-1.27)	<.001	1.23 (1.20-1.27)	<.001
Pfizer-BioNTech vaccine × time since vaccination	1.19 (1.16-1.22)	<.001	1.19 (1.16-1.22)	<.001
Johnson & Johnson–Janssen vaccine × time since vaccination	1 [Reference]	NA	1 [Reference]	NA
Diabetes	NA	NA	1.12 (1.09-1.15)	<.001
Cardiovascular disease	NA	NA	1.18 (1.15-1.21)	<.001
Obstructive sleep apnea	NA	NA	1.13 (1.10-1.16)	<.001
Cancer	NA	NA	1.06 (1.04-1.09)	<.001
COPD	NA	NA	1.17 (1.14-1.20)	<.001
Chronic kidney disease	NA	NA	1.23 (1.20-1.26)	<.001
Liver disease	NA	NA	1.09 (1.05-1.14)	<.001
HIV	NA	NA	1.20 (1.03-1.39)	.02
Obesity	NA	NA	1.11 (1.08-1.15)	<.001
Current or former smoker (vs never smoker)	NA	NA	0.96 (0.94-0.99)	.002

Abbreviations: COPD, chronic obstructive pulmonary disease; NA, not applicable; RR, relative risk; VA, US Department of Veterans Affairs.

^a^ Model 1: age, age squared, sex, race, ethnicity, vaccine type, time since vaccination, and vaccine type × time since vaccination. Reference group is no psychiatric disorders.

^b^ Model 2: model 1 plus obesity status, diabetes, cardiovascular disease including hypertension, obstructive sleep apnea, COPD, cancer, chronic kidney disease, liver disease, HIV, and smoking. Reference group is no psychiatric disorders.

^c^ Other race includes American Indian or Alaska Native, Asian, and Native Hawaiian or Other Pacific Islander.

The presence of any psychiatric disorder diagnosis was associated with an increased incidence of breakthrough infection for both younger (aRR, 1.03; 95% CI, 1.00-1.07; *P* = .03) and older (aRR, 1.10; 95% CI, 1.07-1.13; *P* < .001) patients in age-stratified models adjusting for potential confounders (Table 2). However, results diverged when additionally adjusting for medical comorbidities, obesity, and smoking. In fully adjusted models among younger patients, a psychiatric disorder diagnosis was not associated with incidence of breakthrough infection (aRR, 1.00; 95% CI, 0.97-1.03; *P* = .82). In addition, among younger patients, all individual disorders besides bipolar and psychotic disorders were associated with an increased incidence of breakthrough infection when adjusted for potential confounders, but associations were no longer evident for depressive, posttraumatic stress, and alcohol use disorders when additionally adjusting for medical comorbidities, obesity, and smoking. Conversely, in fully adjusted models among older patients, any psychiatric disorder diagnosis (aRR, 1.05; 95% CI, 1.03-1.08; *P* < .001) and all of the individual disorders apart from posttraumatic stress disorder and alcohol use disorder remained associated with an increased incidence of breakthrough infection. Psychotic disorders were associated with a lower incidence of breakthrough infection among younger patients (aRR, 0.90; 95% CI, 0.82-0.97; *P* = .009) but a higher incidence among older patients (aRR, 1.23; 95% CI, 1.15-1.33; *P* < .001), although only 2.8% of the sample (7326 of 263 697) had a psychotic disorder diagnosis (3.6% [3553 of 97 972] among younger patients and 2.3% [3773 of 165 725] among older patients).

In 1 set of sensitivity analyses, we restricted our sample to 241 705 patients who had completed the full vaccination regimen at least 30 days prior as a more conservative definition of breakthrough cases. Results from these analyses were very similar to the primary models, with larger effect sizes (eTable 2 in the Supplement). In another set of sensitivity analyses, we excluded 6526 patients (2.5%) who received booster doses before the end of our follow-up period and observed results that were very similar to those from our primary models (eTable 3 in the Supplement).

## Discussion

In this retrospective cohort study of 263 697 fully vaccinated VA patients, individuals with psychiatric disorder diagnoses had a higher incidence of SARS-CoV-2 breakthrough infection compared with those without psychiatric disorder diagnoses. In the full sample, these results were robust to adjustment for medical comorbidities, obesity, and smoking. However, the pattern of results varied between older and younger patients in fully adjusted models. Among older patients (≥65 years), all specific psychiatric disorders were associated with an increased incidence of breakthrough infection, with increases in the incidence rate ranging from 3% to 24% in models adjusted for sociodemographic characteristics, vaccine type and timing, medical comorbidities, obesity, and smoking. In contrast, among younger patients (<65 years), there was variability in associations between specific psychiatric disorders and incident breakthrough infections. Although any psychiatric diagnosis and depression, posttraumatic stress, anxiety, adjustment, alcohol use, and substance use disorders were associated with a higher incidence of breakthrough infection in confounder-adjusted models, only anxiety, adjustment, and substance use disorders were associated with an increased incidence of breakthrough infection in fully adjusted models among younger patients. Moreover, psychotic disorders were associated with a 10% lower incidence of breakthrough infection among younger patients. Across younger and older patients, diagnoses of adjustment disorder and substance use disorders were most strongly associated with an increased incidence (9%-24% increase) of breakthrough infection. Although some of the larger observed effect sizes are compelling at an individual level, even the relatively modest effect sizes may have a large effect at the population level when considering the high prevalence of psychiatric disorders and the global reach and scale of the pandemic.

Our data indicate that an increased incidence of breakthrough infection among individuals with psychiatric disorders was not entirely explained by sociodemographic factors, vaccine type or timing, medical comorbidities, obesity, or smoking and that psychiatric conditions may be a risk factor for an increased incidence of breakthrough infection independent of these other factors. Psychiatric disorders (eg, depression, schizophrenia, and bipolar disorders) have been associated with impaired cellular immunity^10^ and blunted responses to vaccines^8,9^; therefore, it is possible that individuals with psychiatric disorders are exhibiting poorer responses to COVID-19 vaccination as well. Evidence has suggested that waning immunity and decrease in vaccine effectiveness against novel variants were associated with breakthrough infections, which is shown in the data on reduced antibody levels over time after vaccination^26^ and lower vaccine effectiveness against new variants.^2,27^ It is possible that immunity wanes more quickly or more strongly among people with psychiatric disorders and/or that they have less protection against newer variants, but this hypothesis needs to be tested. Emerging studies indicate that psychiatric disorders may be associated with increased engagement in risky behaviors for contracting COVID-19,^13,28^ which could also increase the risk for breakthrough infection. Future research should identify immunologic and behavioral mechanisms underlying the heightened risk for SARS-CoV-2 breakthrough infection among individuals with psychiatric disorders to better inform prevention efforts.

Despite the higher overall incidence of breakthrough infection among younger patients, the magnitude of the association between psychiatric disorders and breakthrough infections was generally higher in the older cohort. Moreover, after adjustment for medical conditions, obesity, and smoking, associations between psychiatric disorders and incidence of breakthrough infection were largely attenuated in younger but not older patients, suggesting that some of the excess relative risk of SARS-CoV-2 breakthrough infection associated with psychiatric disorders may be explained by these other factors among younger patients. It is possible that biological or behavioral factors that increase risk for breakthrough infection in individuals with psychiatric disorders are more potent or salient among older patients with psychiatric disorders (eg, poor immunologic response to vaccine^7,8,9,10^ or increased risky behaviors for contracting COVID-19^13^). In other words, the vulnerabilities associated with psychiatric disorders may interact with the vulnerabilities associated with older age to confer greater risk for incident breakthrough infection. Psychotic disorders were associated with a lower incidence of breakthrough infection among younger patients, although the reasons for this are unclear, and younger patients with psychotic disorders comprised our smallest subsample of patients with psychiatric disorders; thus, caution is warranted in interpretating the results. However, these findings are aligned with a study of 51 078 Israeli adults (mean age, 51.5 years) that found that individuals with schizophrenia spectrum disorders had a lower risk for testing positive for SARS-CoV-2, potentially owing to increased testing requirements or increased social isolation.^29^ It is possible that younger individuals with psychotic disorders who managed to complete the vaccination regimen had unmeasured characteristics protecting them against breakthrough infection, but this possibility requires further study. Given that the elevated RR associated with psychiatric disorders was highest among older patients who also experience more severe consequences of infection,^20,21^ older individuals with psychiatric disorders represent a priority group for preventive interventions.

### Limitations

This study has some limitations, including reliance on administrative data and electronic health records, which lack detail and could result in residual confounding or misclassification. We captured breakthrough cases from SARS-CoV-2 test results that were recorded in VA clinical notes from a VA facilty or elsewhere; therefore, we likely underestimated breakthrough cases that were asymptomatic, not tested, or that were assessed elsewhere. The sample included only patients with VA records of vaccination and therefore may underrepresent the full population of veterans who were vaccinated outside of VA facilities without vaccination status recorded in VA clinical records. Patients with psychiatric disorders may access health care more often, resulting in more frequent surveillance testing for SARS-CoV-2.^30^ However, everyone in our analytic sample had accessed VA health care in the past year and had at least 1 documented SARS-CoV-2 test, reducing the concern for selection bias due to health care access. We classified psychiatric disorder diagnoses into broad categories based on *ICD* codes; however, specific individual disorders or specific symptom clusters may have heterogeneous associations with the incidence of breakthrough infection. Given that our sample of US adults who accessed VA health care were mostly male and of older age, our findings may be specific to this population, and additional studies will be needed to ensure that our findings are generalizable to other populations. Moreover, we were unable to adjust for socioeconomic factors, which could be important covariates in associations between psychiatric disorders and breakthrough cases. Our study also did not assess the severity of breakthrough infections. Because psychiatric disorders may increase the risk for more severe COVID-19 sequelae in unvaccinated individuals,^5,6^ future studies should examine whether SARS-CoV-2 breakthrough infections among individuals with psychiatric disorders are associated with worse disease outcomes.

## Conclusions

In this large-scale cohort study of VA patients, psychiatric disorders were associated with an increased incidence of SARS-CoV-2 breakthrough infections, with robust associations among older veterans. In fully adjusted models, individual psychiatric disorders were associated with a 3% to 16% increased incidence of breakthrough infection in our sample, which is comparable to the 7% to 23% increased incidence of breakthrough infection that we observed for physical comorbidities (eg, cancer, kidney disease, and cardiovascular disease). There was variability in the magnitude of the increased incidence associated with specific psychiatric disorders, with larger effect sizes observed for adjustment disorder and substance use disorders among all adults, in addition to adjustment, bipolar, and psychotic disorders among older adults. Psychiatric disorders remained significantly associated with incident breakthrough infections above and beyond sociodemographic and medical factors, suggesting that mental health is important to consider in conjunction with other risk factors. Our findings indicate that individuals with psychiatric disorders may be a high-risk group for COVID-19 and that this group should be prioritized for booster vaccinations and other critical preventive efforts, including increased SARS-CoV-2 screening, public health campaigns, or COVID-19 discussions during clinical care.

## Notes

Supplement. **eTable 1.** *International Classification of Diseases, Ninth Revision, Clinical Modification* (*ICD-9-CM*) and *ICD-10-CM* Codes Used to Define Psychiatric Disorders **eTable 2.** Associations Between Any Psychiatric Disorder and Breakthrough SARS-CoV-2 Infections Among Fully Vaccinated VA Patients at Least 30 Days Postvaccination (n = 241,705), in the Full Sample and Age-Stratified **eTable 3.** Associations Between Psychiatric Disorders and Breakthrough SARS-CoV-2 Infections Among Fully Vaccinated VA Patients Excluding Those With Booster Vaccination (n = 257,171), in the Full Sample and Age-Stratified
