# Supplementary material for: Association of Psychiatric Disorders With Incidence of SARS-CoV-2 Breakthrough Infection Among Vaccinated Adults
Source: JAMA Netw Open. 2022 Apr 14;5(4):e227287. doi: 10.1001/jamanetworkopen.2022.7287 (PMC9011123; doi:10.1001/jamanetworkopen.2022.7287)
Supplement: Supplement. — eTable 1. International Classification of Diseases, Ninth Revision, Clinical Modification (ICD-9-CM) and ICD-10-CM Codes Used to Define Psychiatric Disorders eTable 2. Associations Between Any Psychiatric Disorder and Breakthrough SARS-CoV-2 Infections Among Fully Vaccinated VA Patients at Least 30 Days Postvaccination (n = 241,705), in the Full Sample and Age-Stratified eTable 3. Associations Between Psychiatric Disorders and Breakthrough SARS-CoV-2 Infections Among Fully Vaccinated VA Patients Excluding Those With Booster Vaccination (n = 257,171), in the Full Sample and Age-Stratified [file jamanetwopen-e227287-s001.pdf]

Supplementary Online Content

Nishimi K, Neylan TC, Bertenthal D, Seal KH, O'Donovan A. Association of psychiatric disorders with incidence of SARS-CoV-2 breakthrough infection among vaccinated adults. *JAMA Netw Open.* 2022;5(4):e227287. doi:10.1001/jamanetworkopen.2022.7287

eTable 1. *International Classification of Diseases, Ninth Revision, Clinical Modification* (*ICD-9-CM*) and *ICD-10-CM* Codes Used to Define Psychiatric Disorders
eTable 2. Associations Between Any Psychiatric Disorder and Breakthrough SARS-CoV-2 Infections Among Fully Vaccinated VA Patients at Least 30 Days Postvaccination (n = 241,705), in the Full Sample and Age-Stratified
eTable 3. Associations Between Psychiatric Disorders and Breakthrough SARS-CoV-2 Infections Among Fully Vaccinated VA Patients Excluding Those With Booster Vaccination (n = 257,171), in the Full Sample and Age-Stratified

This supplementary material has been provided by the authors to give readers additional information about their work.

© 2022 Nishimi K et al. *JAMA Network Open.*

eTable 1. *International Classification of Diseases, Ninth Revision, Clinical Modification* (*ICD-9-CM*) and *ICD-10-CM* Codes Used to Define Psychiatric Disorders

Psychiatric Disorder	*ICD-9-CM*	*ICD-10-CM* Codes
Posttraumatic Stress Disorder	309.81 Posttraumatic Stress Disorder	F43.1 Posttraumatic Stress Disorder
Major Depressive Disorder	293.83 Mood disorder in conditions classified elsewhere<br>296.20-296.25 Major depressive affective disorder, single episode<br>296.30-296.35 Major depressive affective disorder, recurrent episode<br>296.82 Atypical depressive disorder<br>300.4 Dysthymic disorder<br>311 Depressive disorder, not elsewhere classified	F32.0-4 Major depressive disorder, single episode<br>F33.0-3 Major depressive disorder, recurrent<br>F33.8 Other recurrent depressive disorders<br>F33.9 Major depressive disorder, recurrent, unspecified<br>F34.1 Dysthymic disorder<br>F34.81 Disruptive mood dysregulation disorder<br>F34.89 Other specified persistent mood disorders<br>F34.9 Persistent mood [affective] disorder, unspecified<br>F39 Unspecified mood [affective] disorder
Anxiety Disorder	300.00 Anxiety state, unspecified<br>300.01 Panic disorder without agoraphobia<br>300.02 Generalized anxiety disorder<br>300.09 Other anxiety states<br>300.20 Phobia, unspecified<br>300.21 Agoraphobia with panic disorder<br>300.22 Agoraphobia without mention of panic attacks<br>300.23 Social phobia<br>300.29 Other isolated or specific phobias<br>300.30 Obsessive-compulsive disorders<br>300.70 Hypochondriasis<br>300.81 Somatization disorder<br>300.82 Undifferentiated somatoform disorder<br>300.89 Other somatoform disorders	F40.00 Agoraphobia, unspecified<br>F40.01 Agoraphobia with panic disorder<br>F40.02 Agoraphobia without panic disorder<br>F40.10 Social phobia, unspecified<br>F40.11 Social phobia, generalized<br>F40.210 Arachnophobia<br>F40.218 Other animal type phobia<br>F40.220 Fear of thunderstorms<br>F40.228 Other natural environment type phobia<br>F40.230 Fear of blood<br>F40.231 Fear of injections and transfusions<br>F40.232 Fear of other medical care<br>F40.233 Fear of injury<br>F40.240 Claustrophobia<br>F40.241 Acrophobia<br>F40.242 Fear of bridges<br>F40.243 Fear of flying<br>F40.248 Other situational type phobia<br>F40.290 Androphobia<br>F40.291 Gynephobia<br>F40.298 Other specified phobia<br>F40.8 Other phobic anxiety disorders<br>F40.9 Phobic anxiety disorder, unspecified<br>F41.0 Panic disorder without agoraphobia<br>F41.1 Generalized anxiety disorder<br>F41.3 Other mixed anxiety disorders<br>F41.8 Other specified anxiety disorders<br>F41.9 Anxiety disorder, unspecified<br>F42.2 Mixed obsessional thoughts and acts<br>F42.3 Hoarding disorder

© 2022 Nishimi K et al. *JAMA Network Open.*

		F42.4 Excoriation (skin-picking) disorder<br>F42.8 Other obsessive-compulsive disorder<br>F42.9 Obsessive-compulsive disorder, unspecified<br>F45.0 Somatization disorder<br>F45.1 Undifferentiated somatoform disorder<br>F45.20 Hypochondriacal disorder, unspecified<br>F45.21 Hypochondriasis<br>F45.22 Body dysmorphic disorder<br>F45.29 Other hypochondriacal disorders<br>F45.8 Other somatoform disorders<br>F45.9 Somatoform disorder, unspecified<br>F63.3 Trichotillomania
Substance Use Disorder	304.00-02 Opioid type dependence<br>304.10-12 Sedative, hypnotic or anxiolytic dependence<br>304.20-22 Cocaine dependence<br>304.30-32 Cannabis dependence<br>304.40-42 Amphetamine and other psychostimulant dependence<br>304.50-52 Hallucinogen dependence<br>304.60-62 Other specified drug dependence<br>304.70-72 Combinations of opioid type drug with any other drug dependence<br>304.80-82 Combinations of drug dependence excluding opioid type drug<br>304.90-92 Unspecified drug dependence<br>305.20-22 Cannabis abuse<br>305.30-32 Hallucinogen abuse<br>305.40-42 Sedative, hypnotic or anxiolytic abuse<br>305.50-52 Opioid abuse<br>305.60-62 Cocaine abuse<br>305.70-72 Amphetamine or related acting sympathomimetic abuse<br>305.80-82 Antidepressant type abuse<br>305.90-02 Other, mixed, or unspecified drug abuse	F11.1 Opioid abuse<br>F11.2 Opioid dependence (excluding F11.21 Opioid dependence, in remission)<br>F12.1 Cannabis abuse<br>F12.2 Cannabis dependence (excluding F12.21 Cannabis dependence, in remission)<br>F13.1 Sedative, hypnotic or anxiolytic abuse<br>F13.2 Sedative, hypnotic or anxiolytic dependence (excluding F13.21 Sedative, hypnotic or anxiolytic dependence, in remission)<br>F14.1 Cocaine abuse<br>F14.2 Cocaine dependence (excluding F14.21 Cocaine dependence, in remission)<br>F15.1 Other stimulant abuse<br>F15.2 Other stimulant dependence (excluding F15.21 Other stimulant dependence, in remission)<br>F16.1 Hallucinogen abuse<br>F16.2 Hallucinogen dependence (excluding F16.21 Hallucinogen dependence, in remission)<br>F18.1 Inhalant abuse<br>F18.2 Inhalant dependence (excluding F18.21 Inhalant dependence, in remission)<br>F19.1 Other psychoactive substance abuse<br>F19.2 Other psychoactive substance dependence (excluding F19.21 Other psychoactive substance dependence, in remission)
Alcohol Use Disorder	303.00-02 Acute alcoholic intoxication in alcoholism<br>303.90-92 Other and unspecified alcohol dependence<br>305.00-02 Alcohol abuse	F10.1 Alcohol abuse<br>F10.2 Alcohol dependence (excluding F10.21 Alcohol dependence, in remission)
Bipolar Disorder	296.00-05 Bipolar I disorder, single manic episode	F30 Manic episode (excluding F304 Manic episode in full remission)

© 2022 Nishimi K et al. *JAMA Network Open.*

	296.10-15 Manic affective disorder, recurrent episode<br>296.40-45 Bipolar I disorder, most recent episode (or current) manic<br>296.50-55 Bipolar I disorder, most recent episode (or current) depressed<br>296.60-65 Bipolar I disorder, most recent episode (or current) mixed<br>296.70 Bipolar I disorder, most recent episode (or current) unspecified<br>296.80 Bipolar disorder, unspecified<br>296-81 Atypical manic disorder<br>296.89 Other bipolar disorders<br>301.13 Cyclothymic disorder	F31 Bipolar disorder (excluding F31.7 Bipolar disorder, currently in remission)<br>F34 Cyclothymic disorder
Psychotic Disorder	295.00-04 Simple type schizophrenia<br>295.10-14 Disorganized type schizophrenia<br>295.20-24 Catatonic type schizophrenia<br>295.30-34 Paranoid type schizophrenia<br>295.40-44 Schizophreniform disorder<br>295.50-54 Latent schizophrenia<br>295.60-64 Schizophrenic disorders, residual type<br>295.70-74 Schizoaffective disorder<br>295.80-84 Other specified types of schizophrenia<br>295.90-94 Unspecified schizophrenia<br>297.00 Paranoid state, simple<br>297.10 Delusional disorder<br>297.20 Paraphrenia<br>297.30 Shared psychotic disorder<br>297.80 Other specified paranoid states<br>297.90 Unspecified paranoid state<br>298.00 Depressive type psychosis<br>298.10 Excitative type psychosis<br>298.20 Reactive confusion<br>298.30 Acute paranoid reaction<br>298.40 Psychogenic paranoid psychosis<br>298.80 Other and unspecified reactive psychosis<br>298.90 Unspecified psychosis	F20.0 Paranoid schizophrenia<br>F20.1 Disorganized schizophrenia<br>F20.2 Catatonic schizophrenia<br>F20.3 Undifferentiated schizophrenia<br>F20.5 Residual schizophrenia<br>F20.81 Schizophreniform disorder<br>F20.89 Other schizophrenia<br>F20.9 Schizophrenia, unspecified<br>F21 Schizotypal disorder<br>F22 Delusional disorders<br>F23 Brief psychotic disorder<br>F24 Shared psychotic disorder<br>F25.0 Schizoaffective disorder, bipolar type<br>F25.1 Schizoaffective disorder, depressive type<br>F25.8 Other schizoaffective disorders<br>F25.9 Schizoaffective disorder, unspecified<br>F28 Other psych disorder not due to a substance or known physiological condition<br>F29 Unspecified psychosis not due to a substance or known physiological condition
Eating Disorder	307.10 Anorexia nervosa<br>307.50 Eating disorder, unspecified<br>307.51 Bulimia nervosa	F50.00-02 Anorexia nervosa<br>F50.2 Bulimia nervosa<br>F50.81 Binge eating disorder<br>F50.89 Other specified eating disorder<br>F50.9 Eating disorder, unspecified
Attention-Deficit Hyperactivity Disorder	314.00 Attention deficit disorder without mention of hyperactivity<br>314.01 Attention deficit disorder with hyperactivity	F90 Attention-deficit hyperactivity disorder
Dissociative Disorder	300.11 Conversion disorder<br>300.12 Dissociative amnesia<br>300.13 Dissociative fugue<br>300.14 Dissociative identity disorder<br>300.15 Dissociative disorder or reaction, unspecified	F44.0 Dissociative amnesia<br>F44.1 Dissociative fugue<br>F44.2 Dissociative stupor<br>F44.4 Conversion disorder with motor symptom or deficit

© 2022 Nishimi K et al. *JAMA Network Open.*

	300.16 Factitious disorder with predominantly psychological signs and symptoms<br>300.19 Other and unspecified factitious illness<br>300.60 Depersonalization disorder	F44.5 Conversion disorder with seizures or convulsions<br>F44.6 Conversion disorder with sensory symptom or deficit<br>F44.7 Conversion disorder with mixed symptom presentation<br>F44.81 Dissociative identity disorder<br>F44.89 Other dissociative and conversion disorders<br>F44.9 Dissociative and conversion disorder, unspecified<br>F48.1 Depersonalization-derealization syndrome
Adjustment Disorder	308.00 Predominant disturbance of emotions<br>308.20 Predominant psychomotor disturbance<br>308.30 Other acute reactions to stress<br>308.40 Mixed disorders as reaction to stress<br>308.90 Unspecified acute reaction to stress<br>309.00 Adjustment disorder with depressed mood<br>309.10 Prolonged depressive reaction<br>309.21 Separation anxiety disorder<br>309.22 Emancipation disorder of adolescence and early adult life<br>309.23 Specific academic or work inhibition<br>309.24 Adjustment disorder with anxiety<br>309.28 Adjustment disorder with mixed anxiety and depressed mood<br>309.29 Other adjustment reactions with predominant disturbance of other emotions<br>309.30 Adjustment disorder with disturbance of conduct<br>309.40 Adjustment disorder with mixed disburbance of emotions and conduct	F43.2 Adjustment disorder<br>F43.8 Other reactions to severe stress<br>F43.9 Reaction to severe stress, unspecified

© 2022 Nishimi K et al. *JAMA Network Open.*

**eTable 2.** Associations Between Any Psychiatric Disorder and Breakthrough SARS-CoV-2 Infections Among Fully Vaccinated VA Patients at Least 30 Days Postvaccination (n = 241,705), in the Full Sample and Age-Stratified				
	**Model 1**		**Model 2**	
	RR (95% CI)	*p-value*	*RR (95% CI)*	*p-value*
Full Sample				
Any Psychiatric Disorder	1.07 (1.05-1.09)	<.0001	1.03 (1.01-1.05)	<.0001
Age <65 (n=89,436)				
Any Psychiatric Disorder	1.04 (1.00-1.07)	0.024	1.00 (0.97-1.04)	0.76
Age ≥65 (n=152,144)				
Any Psychiatric Disorder	1.10 (1.08-1.13)	<.0001	1.05 (1.03-1.08)	<.0001

CI=confidence intervals. RR=relative risk. SARS-CoV-2=severe acute respiratory syndrome coronavirus 2. VA=U.S. Department of Veterans Affairs. Reference group for each model is No Psychiatric Disorders.
Model 1: age, age squared, sex, race, ethnicity, vaccine type, time since vaccination, and vaccine type*time since vaccination.
Model 2: Model 1 plus obese status, diabetes, cardiovascular disease including hypertension, obstructive sleep apnea, chronic obstructive pulmonary disease, cancer, chronic kidney disease, liver disease, HIV, and smoking.

© 2022 Nishimi K et al. *JAMA Network Open.*

eTable 3. Associations Between Psychiatric Disorders and Breakthrough SARS-CoV-2 Infections Among Fully Vaccinated VA Patients Excluding Those With Booster Vaccination (n = 257,171), in the Full Sample and Age-Stratified

	Model 1		Model 2	
	RR (95% CI)	*p-value*	*RR (95% CI)*	*p-value*
Full Sample				
Any Psychiatric Disorder	1.07 (1.05-1.09)	<.0001	1.03 (1.01-1.05)	0.002
Age <65 (n=96,406)				
Any Psychiatric Disorder	1.03 (1.00-1.06)	0.045	1.00 (0.97-1.04)	0.93
Age ≥65 (n=165,765)				
Any Psychiatric Disorder	1.10 (1.07-1.13)	<.0001	1.05 (1.03-1.08)	<.0001

CI=confidence intervals. RR=relative risk. SARS-CoV-2=severe acute respiratory syndrome coronavirus 2. VA=U.S. Department of Veterans Affairs. Reference group for each model is No Psychiatric Disorders.
Model 1: age, age squared, sex, race, ethnicity, vaccine type, time since vaccination, and vaccine type*time since vaccination.
Model 2: Model 1 plus obese status, diabetes, cardiovascular disease including hypertension, obstructive sleep apnea, chronic obstructive pulmonary disease, cancer, chronic kidney disease, liver disease, HIV, and smoking.

© 2022 Nishimi K et al. *JAMA Network Open.*
